# Vasa Vasorum—A Silent Enemy After EVAR: A Case Report and Review of the Literature

**DOI:** 10.3390/clinpract16060117

**Published:** 2026-06-18

**Authors:** Ilias Prentzas, Vasileios Leivaditis, Chrysa Andrikopoulou, Konstantinos Nikolakopoulos, Chrysanthi Papageorgopoulou, Kate Tabaku, Melina Stathopoulou, Zafeiria Papathanassiou, Polyzois Tsantrizos, Francesk Mulita, Konstantinos Katsanos, Spyros Papadoulas

**Affiliations:** 1 Department of Vascular Surgery, General University Hospital of Patras, Patras Medical School, 26504 Patras, Greecekonstantinosn@yahoo.com (K.N.); chrisanthi.papageorg@gmail.com (C.P.); katetabaku@gmail.com (K.T.);; 2 Department of Cardiothoracic and Vascular Surgery, Westpfalz Klinikum, 67655 Kaiserslautern, Germany; vnleivaditis@gmail.com; 3 Department of General Surgery, General Hospital of Eastern Achaia-Unit of Aigio, 25100 Aigio, Greece; chrysa661@gmail.com; 4 Department of Radiology, General University Hospital of Patras, Patras Medical School, 26504 Patras, Greece; papaze74@gmail.com (Z.P.); katsanoskonstantinos@gmail.com (K.K.); 5 Department of Vascular Surgery, General Hospital of Patras “Saint Andrew”, 26504 Patras, Greece; polyzois.tsantrizos@hotmail.com

**Keywords:** abdominal aortic aneurysm, endovascular repair, endoleak

## Abstract

**Background/Objectives:** Type II endoleaks (T2ELs) remain one of the most frequent causes of aneurysm sac enlargement following endovascular abdominal aortic aneurysm repair (EVAR). While embolization may be effective in typical T2ELs with a clearly identifiable feeding vessel, management becomes more challenging when no visible communication with a side branch can be demonstrated. Emerging evidence suggests that hypertrophic vasa vasorum may contribute to aneurysm sac expansion in these atypical cases. We present a case of refractory atypical T2EL treated by open conversion and discuss the potential role of the vasa vasorum network in its pathophysiology. **Case Presentation:** A 77-year-old man presented with lumbar pain ten years after EVAR for a symptomatic abdominal aortic aneurysm. Computed tomography angiography demonstrated progressive aneurysm sac enlargement to 8.5 cm despite three previous translumbar embolization procedures. Multiple areas of contrast pooling were identified within the aneurysm sac, but no clear communication with a feeding side branch was observed. Owing to persistent sac expansion and symptoms, open conversion was performed with partial endograft explantation and reconstruction using a bifurcated PTFE graft. **Results:** After opening the aneurysm sac and evacuating the thrombus, diffuse bleeding was observed from numerous small vascular orifices distributed throughout the inner sac surface. These findings were considered consistent with a prominent vasa vasorum network. Hemostasis was achieved using a combination of figure-of-eight sutures and electrocautery. The postoperative course was uneventful, and the patient was discharged on postoperative day five. Follow-up imaging demonstrated normal graft patency without complications. **Conclusions:** This case supports the hypothesis that an extensive vasa vasorum network may contribute to aneurysm sac expansion in atypical T2ELs and possibly endotension after EVAR. In patients with refractory sac enlargement, open conversion remains a definitive treatment option. Further research is needed to clarify the underlying mechanisms and to explore targeted therapeutic strategies aimed at modulating angiogenesis and vascular remodeling.

## 1. Introduction

### 1.1. Type II Endoleaks After Endovascular Aneurysm Repair

Endovascular abdominal aortic aneurysm repair (EVAR) has become the preferred minimally invasive treatment for abdominal aortic aneurysms (AAAs), offering reduced perioperative mortality compared to open surgical repair [1]. More than 80% of AAAs are treated with EVAR nowadays, a technique which is associated with higher rates of long-term complications and the need for re-interventions, primarily due to endoleaks (ELs) [1,2]. Type 2 ELs (T2ELs), caused by retrograde blood flow from aortic side branches, are the most frequent type of ELs [1,2]. They have been characterized as the Achilles’ heel of EVAR [3]. While approximately 50–70% of T2ELs resolve spontaneously within the first year after EVAR, persistent T2ELs may trigger aneurysm sac expansion [1]. There is a debate regarding treatment in this clinical scenario. Moreover, we should take into consideration that sac expansion and further anatomic alterations may compromise sealing, leading to concomitant type 1 or 3 ELs, which may remain undiagnosed [2,3]. Guidelines recommend embolization of T2ELs when sac expansion exceeds 5 or 10 mm [1,4]. This is technically feasible for typical ELs when there is visible communication of the contrast sac pooling with the culprit side branch, as depicted on computed tomography angiography (CTA) imaging [3]. Conversely, this is not the case for atypical ELs, where open surgery is reserved for expanding aneurysms, as embolization is not technically feasible or is ineffective [5].

We present a patient with a history of EVAR for a symptomatic AAA from 8 years previous. During his routine annual follow-up, he experienced progressive enlargement of the aneurysmal sac. Three sessions of embolization during the last two years were ineffective at halting expansion. The patient presented to the emergency department complaining of lumbar pain, which was identical to the pain he had experienced before EVAR. Open repair offered definite treatment.

### 1.2. Study Rationale

Although type II endoleaks are traditionally attributed to retrograde perfusion from branch vessels such as lumbar arteries or the inferior mesenteric artery, growing evidence suggests that this explanation may not account for all cases of post-EVAR aneurysm sac expansion. In recent years, attention has increasingly focused on atypical type II endoleaks and endotension, conditions in which aneurysm growth occurs despite the absence of a clearly identifiable feeding vessel. Several authors have proposed that a hypertrophic vasa vasorum network and aneurysm wall neovascularization may contribute to these phenomena [3,4,5].

Despite this emerging hypothesis, direct intraoperative observations supporting the role of the vasa vasorum remain scarce. Most published studies rely primarily on imaging findings or indirect evidence, while the macroscopic appearance of the aneurysm sac during open conversion has rarely been described in detail. As a result, the mechanisms responsible for continued sac expansion in atypical type II endoleaks remain incompletely understood.

The present report describes a patient with progressive aneurysm sac enlargement despite repeated embolization procedures and provides detailed intraoperative findings obtained during open conversion. In addition, the available literature regarding the role of the vasa vasorum in atypical type II endoleaks and endotension is reviewed, with particular emphasis on potential implications for future therapeutic strategies.

## 2. Materials and Methods

### 2.1. Case Data Collection

Clinical, operative, and follow-up data were obtained through a review of the patient’s medical records, operative reports, and imaging studies. Preoperative and postoperative computed tomography angiography (CTA) examinations were reviewed to assess aneurysm morphology, sac expansion, and treatment outcomes. Intraoperative findings were documented prospectively during open conversion and supplemented by photographic documentation obtained during the procedure.

### 2.2. Literature Review

To place the present case into context, a focused review of the literature was performed using the PubMed database. The search included publications addressing type II endoleaks, atypical type II endoleaks, endotension, vasa vasorum, aneurysm wall neovascularization, angiogenesis, and open conversion after endovascular aneurysm repair (EVAR). Priority was given to original studies, review articles, and reports investigating the potential role of the vasa vasorum network in aneurysm sac expansion following EVAR. Relevant references cited within selected publications were also reviewed and included when appropriate.

### 2.3. Ethical Considerations

The patient provided informed consent for the surgical procedure and for the anonymous publication of clinical information and imaging findings. All identifying patient information was removed to preserve confidentiality.

## 3. Case Presentation

A 77-year-old male patient presented to the emergency department complaining of lumbar pain. The pain was identical to the pain he had experienced ten years earlier when he had undergone EVAR for a symptomatic AAA, measuring 5 cm in diameter. At that time, EVAR was elected instead of open repair due to patient’s preference. CTA revealed an 8.5 cm AAA with multiple areas of contrast pooling, attributed to atypical T2EL. No clear communication with a feeding side branch was seen (Figure 1A–C). The patient was compliant with yearly follow-up imaging. Unfortunately, during the last 3 years, the aneurysmal sac had increased in size, and three sessions of translumbar embolization for T2EL did not halt aneurysm expansion (Figure 1D,E). Consequently, open conversion was the only feasible option for this symptomatic aneurysm.

Under general anesthesia, a midline laparotomy was performed. After proximal infrarenal and distal common iliac clamping (beyond the iliac limbs of the stent graft), the sac was opened (Figure 2). The endograft was transected at the neck level and removed as the limbs were easily withdrawn, after small longitudinal incisions were made in the common iliac arteries (Figure 3). After evacuation of luminal thrombus, substantial diffuse hemorrhage on the entire expanse of the internal sac surface was found (constituting the most notable intraoperative finding) (Figure 4). We attributed the bleeding to tiny ELs from the ostia of multiple vasa vasorum. Perhaps an extensive network of vasa vasorum surrounding the aneurysm was apparent (Figure 5). Multiple figure-of-eight sutures were placed from within the sac to control the bleeding of larger openings and electrocautery was used for the remaining.

A standard PTFE bifurcated graft (W.L. Gore & Associates, Flagstaff, AZ, USA) was then interposed. Postoperative outcome was uneventful, and the patient was discharged on the 5th postoperative day. Follow-up CTA at 2 months revealed normal graft patency (Figure 6). One year later, he had a normal abdominal aortic colour duplex ultrasound scan. Informed consent has been obtained.

## 4. Discussion

T2ELs are more benign in comparison with the other types of ELs. Sometimes some T2ELs are progressing fast. They are characterized as ‘early’ when they appear in the first month (20–40%). The majority resolve spontaneously, but if they persist beyond 6 months, they are called ‘persisting’ T2ELs. ‘Late’ T2ELs present after 12 months. ‘Refractory’ are the T2ELs that persist after embolization [1]. In case of a stable or shrinking sac, no intervention is needed. Conversely, an expanding sac in the presence of a T2EL raises concerns about potential aneurysm rupture, and special care is required, although the incidence of aneurysm rupture is generally considered low (below 1%) [1,2,6,7,8,9,10].

In a recent study, with a mean follow-up of 4.6 years, it seems that the presence of a T2EL does not affect overall survival. By contrast, it may affect the aneurysm-related mortality (1% vs. 0.2%), aneurysm rupture rates (0.8% vs. 0.1%), likelihood of sac expansion above 5 mm (27.4 vs. 2.7%), reintervention rates (14.9 vs. 0.7%) and the formation of type I or III ELs (1.9% vs. 0.07%), which are implicated in these adverse events [11]. As overall survival is not affected by the T2ELs, there are many concerns about the benefits and the optimal timing of any therapeutic intervention. A universally accepted management algorithm has not been clearly established to date [3]. European guidelines suggest endovascular embolization in the presence of significant sac expansion (>10 mm) compared with baseline or with the smallest diameter during follow-up [1]. However, American guidelines suggest a lower threshold (>5 mm) [2]. The alternative approaches are transarterial, translumbar, transcaval, transabdominal, perigraft, and transgraft techniques [3]. The choice depends on anatomical factors and needs individualization. These methods show high technical success but significant recurrence rates and an inability to halt aneurysm expansion in a significant group of patients [1,12]. Translumbar and transcaval have higher success rates and lower complication rates compared to transarterial [1,13]. A more effective method uses translumbar fusion guided embolisation with needle trajectory planning [3].

In a recent study by Miceli et al., approximately 37% of patients with T2EL refractory to an embolization procedure experienced aneurysm expansion. A total of 42% of these patients underwent open conversion, and the remaining had a second embolization procedure with unsatisfactory results (75% failure). The authors suggest strict follow-up and possibly more aggressive treatment in an elective setting for patients with refractory T2EL [12]. It appears that in cases of atypical T2ELs, the rate of embolization failure is higher. In a recent study by Iwakoshi et al. with 315 patients embolized for T2EL, only 37% achieved freedom from sac expansion at 5 years. The authors claim that the two factors responsible for the worst results are large aneurysms (>7 cm) and atypical T2ELs, which they call ‘Moyamoya ELs’. These are ELs from multiple sac-feeding vessels, including vasa vasorum. This term is derived from the angiographic similarity with Moyamoya disease in the brain [14]. In another study by Vandenbulcke et al., 60 patients were embolized for T2EL. In 52% of these patients, the sac continued to enlarge despite successful embolization. Only 25% had a stable sac without evidence of EL. The authors claim that atypical ELs (which they call ‘Unsharp or blurred T2EL delineation’) are a risk factor for unsatisfactory results. Moreover, 11 patients (18.3%) underwent open conversion, 3 (5%) experienced aneurysm rupture, and 2 experienced graft infection. The authors suggest proceeding with early open conversion in case of atypical T2ELs and post-embolization sac expansion. Interestingly, survival did not differ between patients with or without sac expansion after embolization (21% at 10 years) [15].

Open conversion should be considered for post-embolization refractory T2ELs combined with sac expansion [2]. Surgical options with increasing surgical risk are the following: (a) Simple ligation of side branches without sac opening, (b) Suturing of side-branch orifices from within after sac opening, the so-called “endo-aneurysmorrhaphy”, (c) partial endograft removal to facilitate suturing of these side-branch orifices from within and reconstruction and (d) total endograft removal and replacement with a new graft [16]. The choice between these options depends on the patient’s fitness. In a recent study by Morisaki et al. with 40 patients, endo-aneurysmorrhaphy was compared with endograft explantation. The freedom from aneurysm sac expansion was 76% vs. 100% in these groups, respectively, in 5 years. Thirty-day mortality was 0% in both groups. The authors conclude that more studies are needed to define the most appropriate surgical procedure [17]. Ozaka et al. suggested opening aneurysmorrhaphy without vascular control and strict wrapping over the graft, after excess sac wall removal [18]. Laparoscopic ligation of the inferior mesenteric artery has been used in studies with satisfactory results. In a recent systematic review, technical success was 92% and sac regression was 73% in a 20-month follow-up [19].

Vasa vasorum embolization has been proven futile in the treatment of atypical T2ELs or T5ELs. In a recent study by Takahashi et al., clinical effectiveness was zero, as none of the 7 patients achieved freedom from sac expansion. The authors suggest open conversion instead of vasa vasorum embolization [5]. The role of vasa-vasorum in the pathogenesis of T2ELs or T5ELs has gained increasing interest in recent years [5,20,21]. T5ELs (the so called ‘endotension’ or ‘aneurysm expansion without visible endoleak’ in the current terminology) may share the same pathogenetic mechanisms with atypical T2ELs, but more research is needed focusing on new high resolution imaging methods to delineate sac connections with extremely tiny vasa vasorum [22]. Type 4 endoleaks are not implicated inn sac expansion, as they may take place only during the first month due to transient graft porosity [7].

Vasa vasorum are tiny vessels that constitute a rich network around the arterial wall, contributing to its nourishment by providing blood supply to the outer layers. They are invisible in the normal aortic wall using current imaging modalities. The inner layers of the aortic wall receive oxygen and nutrients through direct diffusion from the lumen. This process is halted after endograft placement, and hypoxic wall conditions may lead to hypertrophy and growth of the vasa vasorum network. Embolization of this network may exacerbate hypoxic wall conditions and promote aneurysm expansion, although complete embolization of this network is generally considered unrealistic [23].

These tiny vessel orifices seen at the inner side of the aneurysm wall could also be an inflammatory response caused by the interaction of the aneurysm wall with the thrombus surrounding the stent graft. Thrombus material contains growth factors and proteolytic enzymes that might also cause an inflammatory response with secondary generation of angiogenesis in the aortic aneurysm wall [20,24,25,26,27,28].

The intraluminal thrombus (ILT) is increasingly recognized as an active contributor to abdominal aortic aneurysm (AAA) progression rather than a simple bystander. By acting as a barrier to oxygen diffusion, the thrombus may create a hypoxic environment within the aneurysm wall [29,30]. Hypoxia has been associated with activation of the HIF-1α pathway and increased expression of vascular endothelial growth factor (VEGF), both of which promote angiogenesis and the development of new vessels arising from the vasa vasorum [30]. These newly formed vessels are often fragile and highly permeable, facilitating the infiltration of inflammatory cells such as macrophages, lymphocytes, and neutrophils into the aortic wall [29,30].

The resulting inflammatory response is accompanied by the release of cytokines and matrix metalloproteinases (MMPs), which contribute to the degradation of elastin and collagen, two key structural components of the aortic wall [29,31,32]. Over time, this process may lead to smooth muscle cell loss, weakening of the vessel wall, and continued aneurysm expansion, ultimately increasing the risk of rupture [29,32].

Novel studies indicate that a hypoxic aortic wall environment after stent-graft placement activates the Hypoxia-Inducible Factor 1-alpha (HIF-1a) signaling pathway [33,34]. This factor subsequently promotes overexpression of Vascular Endothelial Growth Factor (VEGF), which leads to rapid neovascularization. These newly formed vessels are abnormal: they are fragile, permeable, and prone to bleeding, creating a continuous supply of blood within the aneurysmal sac. This mechanism may explain the diffuse bleeding along the entire inner surface of the aneurysmal sac induced by a newly formed, fragile, and complex vascular network. This intense pathophysiological activity suggests that T2EL and T5EL are not merely endoleaks from adjacent vessels but rather VEGF-driven biological processes that feed the aneurysm and drive its sac dilation despite prior interventions [33].

Sac filling concepts are emerging as assisted techniques during EVAR to prevent post EVAR aneurysm sac growth and moreover to aid in sac regression [25,26,27,28,29,30,31,32,33,34,35,36,37]. These techniques give encouraging results [38]. In one study, patients experienced high rates of sac regression, no endoleaks, and no need for reintervention at 1-year follow-up [39].

Moreover, the implications derived from the inflammatory processes are that a new therapeutic window opens, targeting these factors. Until now, the only controlled pharmacological trial for T2EL management tested the tranexamic acid (an antifibrinolytic agent that reduces bleeding), but it showed no clinical success, as tranexamic acid does not interfere with the neovascularization pathway [3].

Pharmaceutical treatments targeting VEGF or modifying the hypoxia/inflammation axis, such as HIF-1a inhibitors, corticosteroids, or non-steroid anti-inflammatory drugs (NSAIDs), have been shown to reduce neoangiogenesis [33,40,41]. There is also evidence that statins are associated with improved neovascularization control and an indirect reduction in VEGF levels [42]. This is particularly interesting since many patients are already on this medication, making future translational research both realistic and promising. The most effective solution emerges from ophthalmology, where for over 15 years, local anti-VEGF injections have been the gold standard for treating pathological neovascularization in conditions such as diabetic retinopathy [41,43]. Applying the same principle by locally injecting anti-VEGF directly into the aneurysmal sac could potentially stop neoangiogenesis “at its source,” offering a definitive solution without the systemic side effects of traditional therapy.

The potential importance of the vasa vasorum network may extend beyond atypical type II endoleaks and endotension. Similar mechanisms of neovascularization, inflammation, and vascular remodeling have been implicated in the progression of atherosclerotic disease, plaque instability, coronary plaque rupture, and peripheral arterial disease. Consequently, further investigation of vasa vasorum biology may not only improve our understanding of post-EVAR sac expansion but may also provide insights into the pathophysiology of other vascular disorders and identify novel therapeutic targets.

In conclusion, treatment of atypical T2ELs (and T5ELs) is not well established when aneurysm expansion occurs. Embolization is associated with high failure rates, and open conversion is acceptable only for patients with sufficient physiological fitness. New prospective studies are urgently warranted focusing on medical treatment. Ongoing pharmacological research will perhaps change the way we manage T2 and T5ELs in the future.

## 5. Future Directions

The findings observed in the present case raise several questions regarding the mechanisms responsible for aneurysm sac expansion after EVAR. Although atypical type II endoleaks and endotension have traditionally been regarded as distinct entities, increasing evidence suggests that they may represent different manifestations of a common biological process involving aneurysm wall hypoxia, inflammation, and neovascularization. A better understanding of these mechanisms may help explain why some patients continue to experience sac enlargement despite apparently successful embolization procedures.

From a diagnostic perspective, current imaging modalities are often unable to demonstrate extremely small vascular communications within the aneurysm wall. Improvements in CTA technology, dynamic imaging protocols, contrast-enhanced ultrasound, and other advanced imaging techniques may provide greater insight into the role of the vasa vasorum network and help identify patients at risk for continued sac growth.

The therapeutic implications are equally interesting. At present, treatment options remain largely mechanical and include embolization or open conversion. However, if angiogenesis and aneurysm wall neovascularization prove to be important contributors to sac expansion, future treatment strategies may also involve pharmacological approaches aimed at modifying these processes. Whether local or systemic therapies can influence aneurysm wall biology after EVAR remains unknown, but this concept deserves further investigation.

Finally, prospective studies correlating imaging findings, intraoperative observations, histopathological analyses, and clinical outcomes would help clarify the significance of the vasa vasorum in atypical type II endoleaks. Such studies may ultimately lead to a more individualized approach to surveillance and treatment after EVAR.

## 6. Limitations

Several limitations should be considered when interpreting the findings of this report. First, this is a single-case observation, and therefore the conclusions regarding the role of the vasa vasorum network in aneurysm sac expansion cannot be generalized to all patients with atypical type II endoleaks or endotension. Second, although extensive bleeding from multiple small vascular orifices was observed intraoperatively, direct histopathological confirmation of their origin was not available. Consequently, the proposed involvement of a hypertrophied vasa vasorum network remains a plausible interpretation rather than definitive proof.

In addition, current imaging techniques were unable to demonstrate the precise vascular communications responsible for the observed sac perfusion. This limitation reflects one of the major challenges in the evaluation of atypical type II endoleaks and highlights the need for further advances in imaging technology.

Despite these limitations, the present case provides rare intraoperative observations obtained during open conversion after failed embolization procedures. Combined with the available literature, these findings contribute to the ongoing discussion regarding the mechanisms responsible for persistent aneurysm sac expansion after EVAR.

## 7. Conclusions

Atypical type II endoleaks and endotension remain among the most challenging complications following EVAR, particularly when aneurysm sac expansion persists despite repeated endovascular interventions. The present case demonstrated extensive diffuse bleeding from multiple small vascular orifices on the inner surface of the aneurysm sac during open conversion, a finding that may support the proposed role of a hypertrophied vasa vasorum network in the pathophysiology of these conditions. Although embolization remains an important treatment option for conventional type II endoleaks, its effectiveness appears limited in selected atypical cases. Open conversion continues to provide a definitive solution for physiologically fit patients with progressive sac enlargement. In addition, emerging aneurysm sac-filling technologies may offer a promising adjunctive strategy to reduce persistent sac perfusion and promote sac stabilization following EVAR. As our understanding of aneurysm wall biology continues to evolve, future advances in imaging, sac-directed therapies, and targeted medical treatments may provide new opportunities for the prevention and management of post-EVAR sac expansion.

## Figures and Tables

**Figure 1:**
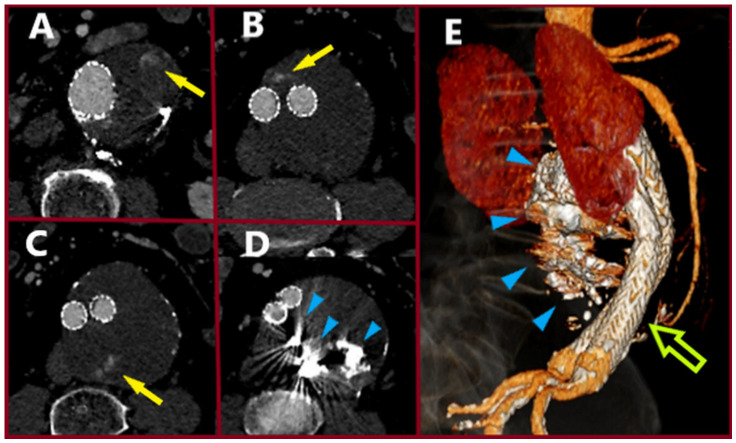
Preoperative Computed Tomography Angiography (CTA). (**A**–**C**) Contrast pooling in the aneurysmal sac due to atypical T2EL in axial images. No clear connection with a feeding branch is detected. Implication of a hypertrophied and developed vasa-vasorum network is considered. (**D**) Onyx embolic material in the aneurysmal sac from previous embolization procedures (blue arrowheads). (**E**) 3D Reconstruction of a CTA depicting the endograft (green arrow) and the embolic material (blue arrowheads).

**Figure 2:**
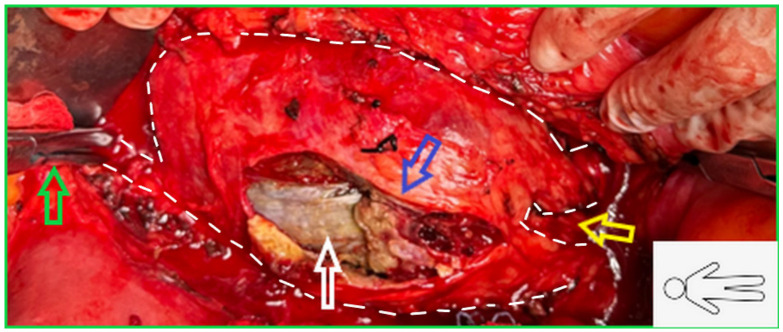
Intraoperative photograph. Following incision of the aneurysm sac at its anterior aspect (blue arrow), the endograft became visible (white arrow). The proximal aortic clamp (green arrow) and the aortic bifurcation (yellow arrow) are also shown.

**Figure 3:**
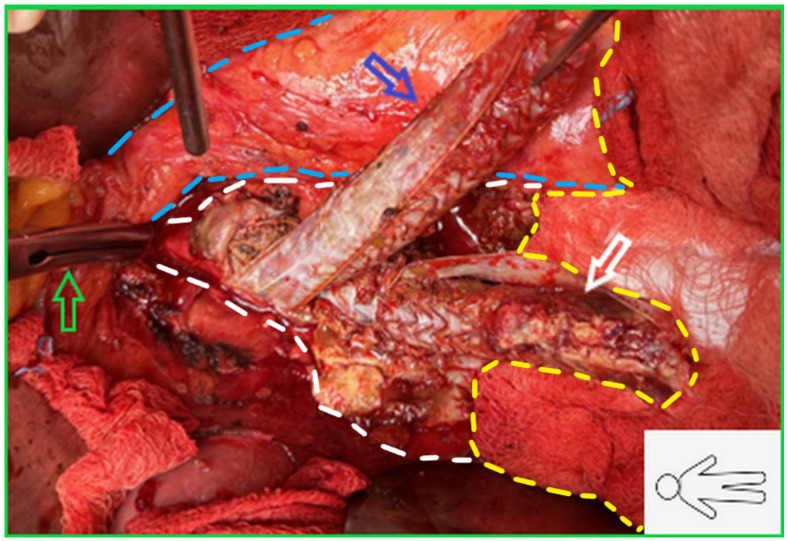
Intraoperative photograph. The left limb of the endograft (blue arrow) has been detached from the left common iliac artery. Subsequently, the right limb (white arrow) is detached from the right common iliac artery. The proximal aortic clamp is also shown (green arrow). The area outlined by the white dotted line represents the proximal portion of the opened aneurysm sac. The distal portion of the sac is covered by surgical gauze and is outlined by the yellow dotted line. The area outlined by the blue dotted line corresponds to the mesentery of the descending colon.

**Figure 4:**
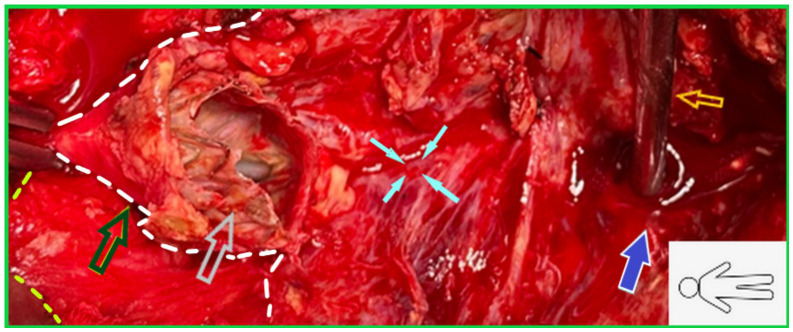
Intraoperative photograph. The proximal body of the endograft (gray arrow) remained in place, while the remainder of the graft was explanted. Significant bleeding (blue arrow) was observed from multiple vasa vasorum orifices on the inner surface of the aneurysm sac following evacuation of the luminal thrombus (green arrow: proximal aortic stump; yellow arrow: suction probe). Most of the image depicts the opened aneurysm sac, outlined by the white dotted line. The vasa vasorum orifices are extremely small and many are obscured by active bleeding; therefore, they cannot be individually identified throughout the image. A larger representative orifice is highlighted in the center of the image by four light-blue arrows. The area between the light-green and white dotted lines corresponds to the duodenum.

**Figure 5:**
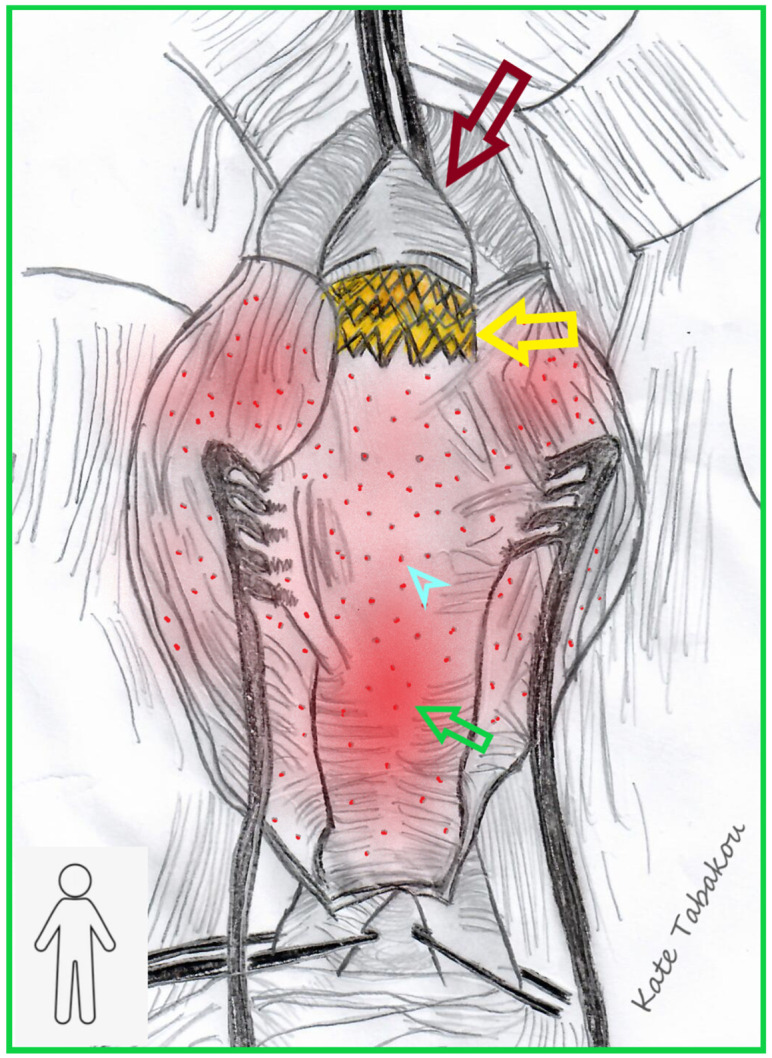
Drawing depicting the intraoperative findings after graft explantation (red arrow: aortic stump, yellow arrow: the remaining proximal part of the endograft, light blue arrow: orifice of a bleeding vasa vasorum, green arrow: blood pooling).

**Figure 6:**
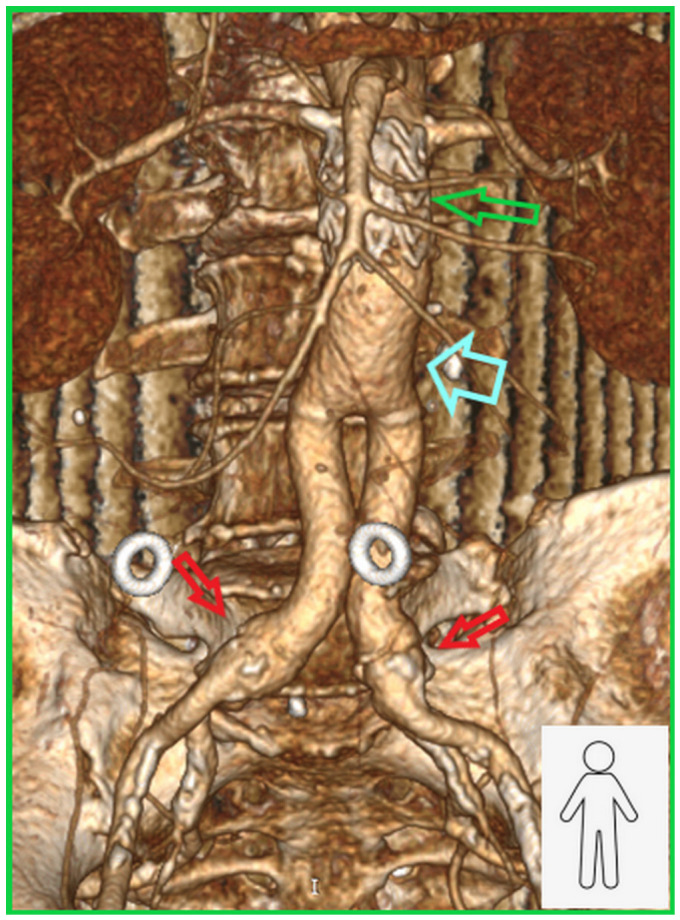
3D Reconstruction of a CTA performed at a 2-month follow-up. It depicts the bifurcated prosthetic graft (blue arrow), the remaining proximal endograft’s body (green arrow), and the distal anastomoses (red arrows).

## Data Availability

The original contributions presented in this study are included in the article. Further inquiries can be directed to the corresponding author.
